# Horizontal return to work coordination was more common in RTW programs than the recommended vertical coordination. The Rapid-RTW cohort study

**DOI:** 10.1186/s12913-019-4607-y

**Published:** 2019-10-26

**Authors:** Lisebet Skeie Skarpaas, Lise Aasen Haveraaen, Milada Cvancarova Småstuen, William S. Shaw, Randi Wågø Aas

**Affiliations:** 1 Presenter – Making Sense of Science, Stavanger, Norway; 2 Department of Occupational Therapy, Prosthetics and Orthotics, Oslo Metropolitan University, Oslo, Norway; 3 Department of Nursing and Health Promotion, Oslo Metropolitan University, Oslo, Norway; 4 Division of Occupational and Environmental Medicine, University of Connecticut School of Medicine, Farmington, CT USA; 5 Department of Public Health, Faculty of Health Sciences, University of Stavanger, Stavanger, Norway

**Keywords:** Return to work, Occupational rehabilitation, RTW intervention, RTW coordination, Rapid RTW- project, Service integration, Sick leave

## Abstract

**Background:**

In return-to-work (RTW) programs, coordinators are often provided in order to integrate services. However, models of coordinating services vary widely internationally, and across different programs, where one distinction is between vertical and horizontal integration (i.e. between levels/institutions, or within one service/level). The aim of this study was therefore to explore and describe if and how a coordinator was provided in RTW-programs, and whether the provision of a coordinator was associated with certain personal or intervention characteristics.

**Methods:**

The study was designed as a cohort study following employees participating in a variety of Rapid-RTW-programs in Norway (*n* = 39). Employees (*n* = 494) answered a self-administered questionnaire, which was linked to register-data on diagnoses and sickness-absence. Employees who replied yes/no to the question “Did the program provide a person who tailored or coordinated your services?” were included in this analysis. Associations for being provided with a coordinator were tested in adjusted logistic regression models.

**Results:**

Sixty-nine percent of the employees reported having a coordinator. These coordinators were mainly responsible for coordinating treatment within own programs (i.e. horizontal coordination, 68%). As expected, rehabilitation programs more often provided a coordinator compared to treatment programs (OR 3.87 95% CI 2.42–6.24). The odds for being provided with a coordinator were reduced for each additional year of age of the employee (OR 0.97, 95% CI 0.96–0.99). More professions were involved in programs that provided coordinators, also more contact with other stakeholders like leaders and social insurance services (NAV), but only contact with supervisor remained statistically significant in adjusted analysis (OR 1.69 95% CI 0.31–9.27). The programs with a coordinator more often provided adaptations at the workplace for the individual employee (OR 0.08 95% CI 0.01–0.60). However, these signs of vertical integration were only evident for a limited number of employees.

**Conclusion:**

In this study, seven of ten employees reported to have a coordinator, which was associated with more professions and stakeholder involvement in the RTW-process. Most of these coordinators did not coordinate vertically between the service levels and types of intervention arenas for sick listed employees (i.e. workplace, social security, and health care services), as recommended in earlier research.

## Background

Internationally there is a trend towards building more integrated health care, focusing on improving the linkages between functions, institutions and professions in the health and social services [1]. This type of integration of services is described as vertical, referring to coordination across various levels and institutions [2, 3]. Another type of organization of services is horizontal integration, which refers to coordination across one level or service [1]. Hvinden (1994) defines coordination as vertical integration [2, 3], while Kärrholm (2007) describes coordination as including both vertical and horizontal integration, with the focus on vertical across levels coordination [2]. Although the aim of integration is to improve coordination and integration of services, the scope of what is to be integrated varies across different services [1]. Today best practice of RTW-programs include social and contextual factors as well as workplace interventions, in a biopsychosocial framework [4–7]. RTW-interventions require cooperation between several stakeholders and across arenas and levels at the workplace, the health care services and the welfare system [6, 8, 9]. Ideally, interventions from these three arenas should be vertically integrated and experienced as one seamless RTW-process for each individual [1, 10].

Several intervention components are found to be essential for facilitating RTW, including centralized coordination of the employees RTW, formal individual psychological and occupational interventions, workplace-based interventions, work accommodations, contact between various stakeholders and interventions to foster concerted action [8, 11, 12]. Facilitation of RTW is hence a complex practice facing several obstacles. One strategy to overcome challenges with integrated care has been to provide a coordinator [1]. Provision of a coordinator has been positively associated with time to RTW in occupational rehabilitation [11, 13–16], and is described as one of the core components for successful return to work [16]. However, a recent review concluded that evidence does not support that RTW-coordination programs that provide a RTW-coordinator promote RTW [17]. The evidence in the review is reported to be of low quality, and more comprehensive studies focusing on sustainable RTW and the workplace are therefore recommended [17]. In contrast, another review concluded that there is strong evidence for recommending service coordination (ex. RTW plans, case management) in multiple component RTW-models together with health-focused and work modification components [18]. RTW is not only an aim for the individual due to health, social and economic reasons, but also for society. The costs of sickness absence and disability are considerable, and RTW-coordination is reported among cost-effective RTW-intervention components [19–21]. Even though there is an ongoing debate on the effect of RTW-coordination and provision of a RTW-coordinator, there is still a continuing need for integration of services and workplace focus in the return to work processes.

Internationally, integration of services is often solved by RTW-coordinators employed by insurers, employers, or governmental agencies [22], with RTW-coordinators being a well-established part of the RTW-process [16]. Reviews of RTW-coordinators revealed the activities of workplace assessment, planning transitions and facilitating stakeholder cooperation with focus on communication and problem solving [16, 22]. Still, a recently published Canadian paper concludes that the integration of services is far below recommendation [23]. Instead, the RTW-coordinators in large companies mostly focused on the employee-supervisor dyad, a horizontal integration, and did not coordinate towards health and welfare services or other stakeholders [23].

In Scandinavia, coordination between stakeholders in RTW-processes is lacking [2, 24–27]. The coordinating agent in RTW is in most cases the social insurance agency, a service separated from health care. However, the responsibility for providing a coordinator is not designated to a specific organization or authority [2]. Although vertical integration of services has been outlined in several policy documents, as the Coordination reform in Norway exemplifies [28], the practice, responsibilities and organizational structures of coordination are still reported to be inadequate [9, 27, 29]. Studies of coordination and provision of a coordinator are often performed in trials where the coordination is provided as a component in RTW-programs [15, 16, 30]. However, few have examined the coordination and cooperation between stakeholders in a real setting with observational design. A Swedish study of Social Insurance agency actions towards employees on long-term sick leave concluded with limited use of both vocational rehabilitation suggestions from the medical assessments, and active rehabilitation measures. Furthermore, of the activities undertaken by the social insurance agency, few actually enhanced RTW [31]. The focus on work rehabilitation and effect on RTW in the Norwegian Social Insurance agencies (NAV) have similarly been questioned [32, 33]. The reform in NAV has actually been found to have a negative impact on RTW [33], and failure to achieve the goal of more people in work seems to be rooted in structural challenges in NAV [32].

As shown above, coordination of services are reported to be inadequate, at the same time coordination and provision of a RTW-coordinator are emphasised as important intervention components in research as well as in policy documents. Accordingly, there is still a need for more comprehensive research in the field of coordination and provision of a coordinator in RTW-processes [17, 34]. Studies on the prevalence of coordinators in RTW-programs, and on predictors for being provided with a coordinator, have to the best of the authors’ knowledge not been published. In Norway, the limited number of guidelines for how RTW-programs should evolve also made it imperative to describe the current model in two levels; the provision or not of a coordinator, and the vertical versus horizontal integration in coordination. We do not know how frequent and to whom a coordinator is provided in RTW-programs, what the coordinator coordinates, or which personal or intervention characteristics impact the provision of a coordinator. Which factors may be associated with some employees being assigned a coordinator but not others? In order to develop RTW-programs in line with best available evidence, it was therefore imperative to explore the prevalence of coordinators, and investigate if there were any patterns in the rapid-RTW-programs’ provision of coordinators.

### Aim

The aim of this study was therefore to explore and describe if and how a coordinator was provided in RTW-programs in Norway, and whether the provision of a coordinator was associated with certain employee, program or intervention characteristics.

## Methods

The study was designed as a longitudinal cohort study of 494 employees participating in Rapid-RTW-programs in Norway.

### Setting

The present cohort-study was one of several studies in the Rapid-RTW-project, an evaluation of the national Rapid-RTW-program in Norway [27], called “Raskere tilbake”. The Rapid-RTW-program is a national program aimed at reducing time to return to work for sick-listed employees or persons at risk of becoming sick listed, and to reduce the waiting-time for specialist assessment and treatment for employees on sick leave. To date, the program is the largest effort for promoting RTW in Norway [27]. Since the program was implemented in 2007, it has had an annual budget of NOK 700 million (approximately $ 85 million USD). The program is organised by the regional specialist health care hospitals and the Norwegian Social Insurance agencies (NAV), and includes more than 200 different public and private RTW-programs. This national program allowed services to respond to tenders in order to get funding to develop and drift RTW-programs, and prioritize patients in a work relation for assessment, treatment and rehabilitation. From 2018 the funding of rapid-RTW-programs was implemented in the hospitals’ annual budgets of funding from the authorities [35]. Each of the rapid-RTW-programs decided the organization, content and intervention components, like the provision of a coordinator. Thus, the local rapid-RTW-programs was not given any instructions from the funding authorities as to whether a coordinator should be assigned. However, only four programs (which gave services to a total of seven participants) have reported, either by the employee or the service provider, not to provide a coordinator to any of its participants. There were generally few requirements for how to implement RTW-programs through the Rapid-RTW-programme resulting in diverse program development [27, 29]. In order to evaluate and develop the program as a whole, it was critical to understand how earlier revealed effective intervention components was implemented in the local rapid-RTW-programmes. This study therefore had the purpose of investigating how the intervention component of coordinator assignment had developed in the RTW-programs.

### Data collection

Each program, clinic or institution offering a rapid-RTW-program was contacted with an invitation to participate in the study. Programs that agreed to participate (*n* = 50) provided a local study coordinator, who recruited participants to the study in the period February to December 2012. Some programs did not manage to recruit personnel to manage the study, and some did not manage to recruit participants, or collect data appropriate, resulting in a total of 46 programs included in this study. Both employees (patients) and their providers answered self-administered questionnaires, including questions about the provision of a coordinator. The questionnaire was developed for this project (Additional file 1), and consisted of both questions developed for this study as well as validated assessments and questionnaires. A total of 679 employees completed the questionnaire. Data on type of service and diagnosis was retrieved from the Norwegian Social Insurance Register (FD-trygd). Data on sickness absence was retrieved from the Norwegian Social Insurance Register. The register data was linked to the self-reported data using an eleven-digit personal identification number. Participants who replied yes/no to the question “Did the program provide a person who tailored or coordinated your services?” were included in this analysis. Those who answered “I do not know” (*n* = 120) or did not answer this question (*n* = 65) were excluded.

### Participants

See Table 2 for participants’ characteristics. In total, 134 males and 360 females (total *n* = 494) from 46 different institutions were included in the present study. The participants’ median age was 46 years (min-max. 21–70), and the majority had a history of sickness absence i.e. been on sick leave on at least one earlier occasion (96%). The most common diagnoses were musculoskeletal problems (55%) and mental health problems (16%). Occupational rehabilitation was the most common type of Rapid-RTW-programs, and 57% of the informants received such programs. These programs included rehabilitation in hospitals and institutions, both inpatient and outpatient [27]. Furthermore, 36% of the participants received medical or psychological treatment, including assessment counselling, and surgery (exp. to shorten waiting time for employees on sick leave in need for surgery was a part of the rapid-RTW-program, but are not very common) which were the second most common type of RTW-program provided.

### Statistical analyses

Continuous variables were described using median (range), categorical variables with counts and percentages. Unadjusted associations were assessed using Mann-Whitney-Wilcoxon and Chi-square tests for continuous and categorical variables, respectively. Multiple logistic regression models were fitted to identify adjusted associations between the dependent variable (provision of a coordinator vs no provision) and the independent variables (gender, age (years), marital status (live alone/ with partner), sickness absence before receiving RTW-program (days), diagnosis (MSD/ mental disorders/ cancer/ other diagnosis), self-reported symptoms as experienced at start of program (pain at rest, pain in activity, depressive mood, and anxiety), and educational level (elementary or upper secondary school (up to 12 years)/ university degree). Variables with a *p*-value =/< 0.2 in the univariate analyses were entered into a multiple logistic regression model, and the results are presented as odds ratio (OR), with 95% confidence intervals (CI).

*P*-values < 0.05 were considered statistically significant. All tests were two-sided. All analyses were performed using IBM SPSS Statistics 24.

## Results

In total, 68% of the participants (*n* = 335) reported that they were provided with a coordinator. As shown in Table 1, the coordinators were most often provided by the RTW-program (69%, *n* = 156), meaning the coordinator’s role was managed by one of the professionals involved in the RTW-program. Furthermore, the coordinators were mostly responsible for coordinating their own programs (68%, *n* = 186), and to a lesser extent other services or stakeholders (see Table 1).

**Table 1:** Frequencies of which services provided the coordinator and which services the coordinator did coordinate

Type of service or stakeholder	Yes: this service provided a coordinator n (%)	Yes: this service was included in the coordinators’ coordination n (%)
The Rapid-RTW program	156 (69)	186 (68)
Specialist health care	7 (4)	15 (6)
General practitioner	1 (0.5)	15 (6)
Community health care	1 (0.5)	2 (1)
Workplace	4 (2)	21 (8)
Social Insurance (NAV)	10 (5)	23 (9)
Occupational Health Services	1 (0.5)	8 (3)
Other service	4 (2)	9 (4)

Note: n (%) = number of participants (percentage of participants) that was provided with a coordinator from the different services, and n (%) got the different services or stakeholders included in the coordination by the coordinator

### Personal characteristics associated with being provided with a coordinator

There were no statistical significant differences between those who were provided with a coordinator, compared to those who were not, concerning gender, social status, educational level, or history of sickness absence except for age. See Table 2 for an overview of personal characteristics and provision of a coordinator. The employee’s age was associated with provision of a coordinator. The median age was lower for those provided with a coordinator compared to those not provided with a coordinator, 45 versus 47 years respectively (*p* = 0.01). In the adjusted analysis, the odds for being provided with a coordinator were reduced for each additional year of age of the employee (OR 0.97, 95% CI 0.96–0.99).

**Table 2:** Personal characteristics associated with being provided with a coordinator

Variable	Category	Total n (%)	With coordinator n (%)	Without coordinator n (%)	*p*-value
Gender n (%)	Women	360 (72.9)	248 (74.0)	112 (70.4)	0.42
	Men	134 (27.1)	87 (26.0)	47 (29.6)	
Age median (min-max)		46 (21–70)	45 (21–66)	47 (21–70)	0.01*
Social status n (%)	Live alone	112 (23.2)	78 (23.9)	34 (21.7)	0.58
	Live with others	371 (76.8)	248 (76.1)	123 (78.3)	
Educational level n (%)	Elementary school (up to 9 years)	49 (10.1)	31 (9.5)	18 (11.4)	<0.01**
	Upper secondary school (12 years)	211 (43.4)	149 (45.4)	62 (39.2)	
	University degree (up to 4 years)	153 (31.5)	111 (33.8)	42 (26.6)	
	University degree (> 4 years)	73 (15)	37 (11.3)	36 (22.8)	
Diagnosis n (%)	MSD	270 (54.8)	198 (59.3)	72 (45.3)	<0.01**
	Mental disorders	80 (16.2)	46 (13.8)	34 (21.4)	
	Cancer	43 (8.7)	22 (6.6)	21 (13.2)	
	Other disorders incl. Neuro- and heart diseases	52 (10.5)	40 (12.0)	12 (7.5)	
	Common or unspecific disorders	21 (4.3)	13 (3.9)	8 (5.0)	
	No or missing diagnosis	27 (5.5)	15 (4.5)	12 (7.5)	
Symptoms	Pain at rest	397 (84.5)	282 (88.1)	115 (76.7)	<0.01**
	Pain in activity	414 (88.8)	290 (91.2)	124 (83.8)	0.02*
	Depressive mood	373 (78.9)	252 (79.0)	121 (78.6)	0.92
	Anxiety	285 (60.1)	193 (59.6)	92 (61.3)	0.72
History of sickness absence	Yes	473 (95.7)	324 (96.7)	149 (93.7)	0.12
Sickness absence before RTW-program *N* = 433 median days (range)		147 (0–935)	159 (0–802)	119 (0–935)	0.04*
Sick-leave baseline n (%)					<0.01**
	Full-time (100%)	326 (66.1)	237 (71.0)	89 (56.0)	
	Part-time (20–90%)	105 (21.3)	72 (21.6)	33 (20.8)	
	Not on sick-leave	65 (12.6)	25 (7.5)	37 (23.3)	

Notes: Significance level: * < .05. ** < .01

Almost half (43%) of the employees reported upper secondary school (12 years of schooling) as their highest educational level. There was no statistical difference between those provided with and those not provided with a coordinator (neither unadjusted nor adjusted results) when comparing low and high educational levels. See Table 3 for employee-related factors associated with having a coordinator.

**Table 3:** Employee-related factors associated with having a coordinator

	Unadjusted results			Adjusted results		
Variable	OR	95% CI	*p*-value	OR	95% CI	*p*-value
Age	0.97	0.96–0.99	<0.01*	0.97	0.95–1.00	0.03*
Gender						
Women	1.20	0.79–1.82	0.40	1.030	0.62–1.71	0.91
Men (ref)						
Educational level						
Elementary or Upper secondary school (up to 12 years)	1.19	0.81–1.73	0.38	1.27	0.80–2.02	0.32
University degree (ref)						
Diagnoses						
MSD	1.76	1.20–2.58	<0.01*	1.51	0.92–2.47	0.11
Other diagnoses (ref)						
Pain at rest	2.26	1.36–3.75	<0.01*	2.01	0.77–5.23	0.15
Pain in activity	2.01	1.12–3.60	0.02*	0.96	0.32–2.89	0.94
Sickness absence days before RTW-program	1.00	1.00–1.00	0.05*	1.00	1.00–1.00	0.03*
Sick-leave at baseline						
Full-time (100%)	1.91	1.30–2.85	<0.01*	1.06	0.63–1.79	0.82
Part-time (0–90%) (ref)						

Notes: *Statistical significant at level =/< 0.05

Diagnosis was statistically significant associated with the provision of a coordinator, compared to not being provided with a coordinator. The highest proportion of employees who were referred to a RTW-program were those diagnosed with Musculoskeletal disorders (MSD) (55%). Employees with MSD were 1.8 times more likely to be provided with a coordinator compared to employees with other diagnoses in the unadjusted analysis (OR 1.76, 95% CI 1.20–2.58). However, this association did not remain statistically significant in the adjusted analysis. Regarding symptoms, both depressive mood and anxiety were not associated with higher odds for being provided with a coordinator, compared to not being provided with a coordinator. Employees who reported having pain were twice as likely to be provided with a coordinator compared to those who did not report pain, OR 2.26 (95% CI 1.36–3.75) and 2.01 (95% CI 1.12–3.60) for those with pain at rest and pain in activity, respectively. However, neither pain at rest nor pain in activity remained statistically significant in the adjusted analyses.

Nearly all participants (96%) had a history of sickness absence during the last three years prior to participation in the program. There was statistically significant differences between those provided with and those not provided with a coordinator related to days of sickness absence before the RTW-program started, and related to being on sick leave at baseline (RTW-program start). Those provided with a coordinator had been on sick leave for more days (median 159 days) before the RTW-program compared to those not provided with a coordinator (median 119 days), and this association remained statistically significant in the adjusted analysis. The odds for having a coordinator for employees on sick leave (100%) compared to those not on sick leave or on graded sick leave did not remain statistically significant in adjusted analysis (OR 1.06 95% CI 0.63–1.79).

### Program predictive factors for being provided with a coordinator

There was a statistically significant difference between those provided with a coordinator versus those who were not regarding the type of RTW-program provided. See Table 4 for program characteristics and provision of a coordinator. Employees who received “Occupational rehabilitation” and “Follow-up and Work clarification” were more often provided with a coordinator, compared to those not provided with a coordinator. The odds for being provided with a coordinator when receiving “Occupational rehabilitation” were almost four times higher compared to such odds for “Treatment inclusive assessment and surgery” (OR 3.87 95% CI 2.42–6.24). This association remained statistical significant in the adjusted analysis.

**Table 4:** Program characteristics and provision of a coordinator (employees, n and %)

Variable		Total	With coordinator n (%)	Without coordinator n (%)	*p*-value
Type of intervention n (%)					<0.01*
	Occupational rehabilitation	275 (56.7)	221 (67.0)	54 (34.8)	
	Medical or psychological treatment, including assessment, and surgery	172 (35.6)	77 (23.3)	95 (61.3)	
	Follow-up and Work clarification programs through NAV	38 (7.8)	32 (9.7)	6 (3.9)	
Professionals involved n (%)	Medical doctor	301 (85.0)	216 (88.5)	85 (77.3)	<0.01*
	Physical therapist	299 (83.3)	226 (90.8)	73 (66.4)	<0.01*
	Nurse	177 (56.9)	128 (58.4)	49 (53.3)	0.40
	Nutritionist	171 (54.1)	132 (58.9)	39 (42.4)	<0.01*
	Others	164 (50.6)	121 (52.8)	43 (45.3)	0.21
	Psychologist	141 (42.5)	91 (39.2)	50 (50.0)	0.07
	Vocational consultant	139 (42.4)	109 (47.6)	30 (30.3)	<0.01*
	Social worker	127 (39.0)	91 (40.1)	36 (36.4)	0.53
	Occupational therapist	91 (28.5)	72 (31.7)	19 (20.7)	0.05*
	Pedagogue	88 (31.4)	77 (37.7)	11 (14.5)	<0.01*
	Work instructor	42 (13.2)	30 (13.5)	12 (12.5)	0.84
Provision of a coordinator from other services n (%)	Social Insurance (NAV)^	24 (7.1)	20 (8.5)	4 (3.9)	
	Workplace^	3 (0.9)	3 (1.3)	0 (.0)	
	Occupational Health Services ^	3 (0.9)	3 (1.3)	0 (.0)	
	Others^	3 (0.9)	3 (1.3)	0 (.0)	
	General Practitioner^	2 (0.6)	2 (0.9)	0 (.0)	
	Specialized health care^	1 (0.3)	0 (.0)	1 (1.0)	
	Community based health care^	0 (.0)	0 (.0)	0 (.0)	
Contact with other instances n (%)	General Practitioner	191 (90.5)	149 (92.0)	42 (85.7)	0.19
	Social Insurance consultant (NAV)	116 (81.7)	96 (84.2)	20 (71.4)	0.12
	Leader/supervisor	76 (71.0)	63 (75.9)	13 (54.2)	0.04*
	Specialized health care^	19 (33.9)	15 (36.6)	4 (26.7)	
	Others^	14 (26.9)	10 (27.0)	4 (26.7)	
	Occupational Health Services ^	8 (16.7)	7 (19.4)	1 (8.3)	
	Family^	8 (17.0)	4 (12.1)	4 (28.6)	
	Community based health care^	7 (14.9)	2 (6.5)	5 (31.3)	
	Work-life center (NAV arbeidslivssenter) ^	6 (13.6)	3 (9.7)	3 (23.1)	
Adaptions n (%)	No adaptations were performed	234 (84.5)	149 (78.4)	85 (97.7)	<0.01*
	Work time^	49 (48.5)	48 (58.5)	1 (5.3)	
	Work tasks^	30 (33.0)	27 (38.6)	3 (14.3)	
	Leisure activities^	23 (28.0)	19 (30.6)	4 (20.0)	
	Physical work environment^	17 (20.2)	16 (24.6)	1 (5.3)	
	Psychosocial work environment^	6 (7.6)	6 (9.8)	0 (0.0)	
	Home^	4 (5.1)	3 (5.0)	1 (5.3)	

Notes: All variables except type of RTW-program does not sum to 100% in total and each group with/without a coordinator since employees may have been provided with several or none. ^No statistical tests performed due to insufficient n of individuals. *Statistical significance set at level =/< 0.05

The RTW-programs provided the coordinator in most cases. However, a few participants were provided with a coordinator from other programs, where NAV was the second largest provider of coordinators (7%).

In the programs that provided coordinators, more contact with other stakeholders (i.e. general practitioner, NAV and leader/supervisor) was reported, compared to the programs that did not provide a coordinator. However, only having “contact with supervisor” was statistically significant for those provided with a coordinator compared to those not provided with a coordinator, but this association did not remain statistically significant in the adjusted analysis (OR 1.69 95% CI 0.31–9.27). See Table 5 for program characteristics associated with being provided with a coordinator.

**Table 5:** Program characteristics* associated with being provided with a coordinator

	Unadjusted results			Adjusted results		
Variable	OR	95% CI	*p*-value	OR	95%CI	*p*-value
Age	0.97	0.96–0.99	<0.01*	0.97	0.95–0.99	0.01*
Gender						
Women	1.20	0.79–1.82	0.40			
Men (ref)						
Type of program						
Occupational rehabilitation	5.05	3.31–7.71	<0.01*	3.87	2.41–6.24	<0.01*
Follow-up and Work clarification programs (NAV)	6.58	2.62–16.55	<0.01*	4.77	1.83–12.44	<0.01*
Treatment incl. Assessment and surgery (ref)			<0.01*			<0.01*
Professionals involved						
Medical doctor	2.27	1.25–4.11	<0.01*	1.81	0.84–3.89	0.13
Vocational consultant	2.09	1.27–3.45	<0.01*	1.61	0.78–3.34	0.20
Nutritionist	1.95	1.19–3.19	<0.01*	1.52	0.79–2.93	0.21
Physical therapist	4.98	2.78–8.93	<0.01*	4.75	1.82–12.41	<0.01*
Occupational therapist	1.79	1.00–3.18	0.05*	2.58	1.21–5.50	0.02*
Psychologist	0.65	0.40–1.04	0.07			
Pedagogue	3.58	1.78–7.21	<0.01*	2.02	0.85–4.81	0.11
Adaptations						
No adaptations	0.09	0.20–0.36	<0.01*	0.08	0.01–0.60	0.01*
Contact with other instances						
Leader/supervisor	2.67	1.03–6.88	0.04*	1.69	0.31–9.27	0.54

Notes: Statistical significance set at level =/< 0.05. *p-level < 0.2 in association testing, see Table 4. **Controlled for age, gender, diagnosis, sickness absence days before RTW-program, and type of program

Furthermore, the employees with a coordinator received more adaptations at the workplace. Programs providing a coordinator were more likely to make adaptations in their intervention: It was about 90% less likely that the answer to the question “Did this program provide one of the following types of adaptations?” were “No adaptations were performed” for employees provided with a coordinator, compared to those not provided with a coordinator (OR 0.08 95% CI 0.01–0.60). This association remained statistically significant in the adjusted analysis.

In general, employees provided with a coordinator met more professions in the RTW-programs. The association between those provided with, compared to those not provided with a coordinator was statistically significant related to medical doctor, vocational consultant, occupational therapist, nutritionist, physical therapist and pedagogue. Meeting a psychologist was more common in the group without a coordinator compared to those with a coordinator, however, this association was not statistically significant. In this study, the odds for being provided with a coordinator when having a physical therapist in the program were more than four and a half times higher compared to not having a physical therapist in the program (OR 4.75, 95% CI 1.82–12.41). This association remained statistically significant in the adjusted analysis.

## Discussion

The aim of this study was to explore and describe if and how a coordinator was provided in RTW-programs in Norway, and whether the provision of a coordinator was associated with certain personal or intervention characteristics. Our main findings were; (1) about two-thirds of the employees were provided with a coordinator by the RTW-program, most often coordinating their own programs, (2) younger age and length of sickness absence were predictors for being provided with a coordinator, (3) occupational rehabilitation programs provided a coordinator more often than the other types of RTW-programs, (4) more professions were involved, and there was more contact with other stakeholders and instances outside their program when the employee had a coordinator, and (5) adaptations to the workplace were more common for those provided with a coordinator. These findings will be discussed below.

### Current coordinator practices is mainly horizontal integration

Two out of three employees who received services from the Rapid-RTW-programs were provided with a coordinator offered by the program. The coordinators were mainly responsible for coordinating their own program. Such linking of programs at the same level is referred to as horizontal integration [1]. Thus, the coordinator model revealed in the present study was based on horizontal integration. This is despite the government’s effort for implementing a coordination reform focused on offering comprehensive and continuous services [28], so-called vertical integration. In this perspective, the government expects RTW-programs to cooperate and coordinate their services across stakeholders and arenas. If such practices were evident in the RTW-programs, one could expect the coordinators to be a part of this. However, a study of RTW-coordinators in large companies in Canada also revealed that the coordinators mainly focused on the employee-supervisor dyad, in other words, horizontal integration within the same company [23]. In the present study, the coordinators were most often employed by the RTW-programs, and they coordinated their own services. Even though the literature on RTW and coordination repeatedly calls for more vertical integration [17, 18, 29, 30, 36], this seems to not be implemented in practice.

Being young was a predictor for being provided with a coordinator. One reason for this association might be that younger employees with sickness absence at risk for disability pension have more complex health problems or diagnosis, such as severe mental health problems [37], indicating a need for coordination of services. Furthermore, young people might be prioritized in these services and by society, as they will contribute to society if they return to work with i.e. paying taxes throughout their working life in contrast to becoming a disability pension receiver throughout their lifetime [9]. The finding that older age was associated with reduced odds for being provided with a coordinator is in line with earlier studies [11, 38, 39]. Older age is a strong predictor for delayed return to work [40, 41]. Taking into account the global challenge of an aging work force, interventions aimed at RTW and keeping employees in their job despite health problems is an important field of practice and research [9, 39, 41]. RTW-programs should therefore ensure that they meet the needs of different age groups [38] and provide enough resources and attention in order to support older employees in their RTW-process [39].

Employees with MSD constituted the majority of this study’s participants, and provision of a coordinator was most frequent for employees with MSD in the unadjusted analysis. The effect of provision of a coordinator is also best documented for this group of sick-listed employees [17, 34], although the effects are debated [17]. Employees with a MSD diagnosis will often recover without interventions. Wynne-Jones et al. (2014), for example, found that approximately 70% of employees on sick leave with back pain returned to work within a month [42]. However, those referred to a RTW-program in the present study had an average of more than 5 months of sickness absence, although early intervention to support RTW is recommended [43]. Hence, some will argue it takes too long to be referred to RTW-programs [29]. The timing of when to refer to a RTW-program and provide a RTW-coordinator is highly relevant to discuss. Length of sickness absence before starting the RTW-program was associated with provision of a coordinator in the present study. Delayed return to work is a risk factor for permanent work disability [44], and provision of RTW-coordinators is one intervention component provided in order to enhance timing of programs and planning of the RTW-transition [16]. In addition, an explanation for being provided with a coordinator may be the complex situation associated with long-term sickness absence due to pain and musculoskeletal health problems [45]. Comorbidity is one issue [45], as well as the fact that long-term absence may be a barrier for RTW in itself [9]. In the present study, pain was associated with being provided with a coordinator in the unadjusted analysis. Pain is not only associated with MSD, but also depression and anxiety, and has been revealed to be a strong predictor for disability pension [46]. These factors may call for multiple interventions with several involved stakeholders, and provision of a coordinator will facilitate such an integrated RTW-process.

Employees provided with a coordinator often received occupational rehabilitation programs and had been on sick leave for a longer period before the RTW-intervention. It seems reasonable that those with long-lasting problems are offered more comprehensive interventions with more professionals involved. However, provision of such comprehensive interventions versus brief interventions is debated [47]. It seems some groups benefit more from multiprofessional interventions with several components [43, 48], and some will return to work more rapidly when provided a single brief intervention [47]. In programs with several professionals, it is likely that internal, horizontal coordination or collaboration is necessary, as revealed in present study.

### Signs of vertical integration

Although the coordinator model revealed in Rapid-RTW-programs builds mainly on horizontal integration, some signs of vertical integration in the coordinator practices were found. Some of the intervention components offered in the RTW-programs with a coordinator were associated with factors reflecting vertical integration. More professionals were involved in RTW-programs that provided a coordinator. Multiprofessional involvement is a characteristic of the occupational rehabilitation program [27], and is a predictor for RTW for some employees on sick leave as discussed above [43, 48]. Furthermore, one might reason that comprehensive interventions would require more coordination with stakeholders, both horizontal and vertical. The results show that the aim of coordination to integrate programs across levels and institutions in a vertical manner was met for some of the employees in the present study. Those provided with a coordinator reported more contact with other stakeholders and instances, like leaders and NAV. This may be viewed as signs of vertical integration, which is considered a predictor for RTW in previous studies [8, 15, 18, 43]. However, the coordinator in the Rapid-RTW-programs was reported to mainly coordinate their own programs, and it seems the vertical integration as such was lacking in most cases.

The odds of being offered adaptations was improved for those provided with a coordinator in the present study. Employees who had a coordinator were generally offered more adaptations, including adaptations in the work environment, work time and work tasks. Accommodations at and contact with the workplace has earlier been revealed as success factors for RTW [11, 12, 18]. Furthermore, closer contact with the workplace are described as a way forward in development of intervention components in RTW-programs [17]. Such contact and facilitation of accommodations at the workplace are described as typical activities for RTW-coordinators [16]. Still, only approximately 10% of the employees in the present study were offered adaptations at the workplace, and one might wonder if adaptations to facilitate RTW were an underused intervention component.

### Limitations

For some of the variables the proportion of missing data was high, and this of course lowers the quality of the results for these variables. Consequently, some variables were not included in statistical testing due to low n, for instance the different types of adaptations, provision of a coordinator from other instances, and some of the categories of contact with other instances. Hence, this needs to be further explored and tested in future studies. Even though the difference between having a physical therapist versus not in the program remained statistical significant in adjusted analysis, the confidence intervals were wide, and therefore these results should be replicated to validate their significance. Additional knowledge of the coordinator like their education, profession etc. and how they were distributed would provide valuable insight to the study, as the background of the coordinator has previously been reported to be associated with intensity of engagement and activities the coordinator is involved in [23]. In this study there was no information available on why some employees were provided with a coordinator and some not. In addition, a relatively large number of employees (*n* = 185) were excluded based on missing or unreliable information on the provision of a coordinator, and this could be a weakness. It might be that a large proportion of those excluded did not have a coordinator, however it might also be that the provision of a coordinator was not well communicated when the coordination was internally oriented. The main focus in the current study has been on the provision of a coordinator, and additional information on coordination provided without involvement of a coordinator could have made the total picture of the coordination practices richer. However, the question of contact with other stakeholders etc. was not limited to the coordinator, but involved the whole programs’ practice. Although the analyses show that it might be that severity or complexity (i.e. pain and length of sickness absence) explains some of coordinator distribution on the individual level, the relationship does not remain statistically significant in the adjusted analysis. Studies with more information on complexity or severity of injury, as well as on the individual programs’ criteria for provision of a coordinator should be performed. In addition, the coordinator’s competencies and activities, i.e. contact with the workplace should be further explored in future research. Furthermore, the sample in this study was exclusively from Rapid-RTW-programs, and it might be that other RTW-programs differ in their RTW- and coordination of RTW-models. On the other hand, the Rapid-RTW-program is the largest effort to promote RTW in Norway and therefore the sample is generally representative of RTW-programs provided to sick listed employees in this country.

## Conclusions

Our results revealed that it is common to provide a coordinator in the Rapid RTW-programs in Norway. However, the coordinators for the most part coordinate their own programs, and to a limited degree integrate services vertically across stakeholders, levels and providers. Employees in occupational rehabilitation programs are, in this study, those most likely to be provided with a coordinator. Provision of a coordinator is associated with more involvement of different professions in the program, more contact with other services and more adaptations in regard to the program and the workplace. However, only few experience vertical integration of services in Rapid-RTW-programs. The model of RTW-coordination and provision of a coordinator should be further developed. To distinguish between internal and single level horizontal integration and vertical across levels and stakeholders integration could be one way to test different models’ effects on RTW when providing a coordinator in RTW-programs in the future.

## Supplementary information

**Additional file 1:** Questionnaire for patient and providers in Rapid-RTW-programs. The questions used in analysis to explore and describe if and how a coordinator was provided in RTW-programs in Norway, and whether the provision of a coordinator was associated with certain employee, program or intervention characteristics included in the file.

## Data Availability

The datasets analysed during the current study are not publicly available due to research ethical considerations, but are available from the corresponding author on reasonable request.

## Abbreviations

MSD: Musculoskeletal disorders
NAV: Norwegian Social Insurance agencies
NSD: Norwegian Centre for Research Data
RTW: Return to Work
