# Supplementary material for: Horizontal return to work coordination was more common in RTW programs than the recommended vertical coordination. The Rapid-RTW cohort study
Source: BMC Health Serv Res. 2019 Oct 26;19:759. doi: 10.1186/s12913-019-4607-y (PMC6815375; doi:10.1186/s12913-019-4607-y)
Supplement: Supplementary file 1 — Additional file 1: Questionnaire for patient and providers in Rapid-RTW-programs. The questions used in analysis to explore and describe if and how a coordinator was provided in RTW-programs in Norway, and whether the provision of a coordinator was associated with certain employee, program or intervention characteristics included in the file. [file 12913_2019_4607_MOESM1_ESM.docx]

Additional file 1. Questionnaire for patient and providers in Rapid-RTW-programs.

**Questions for patients:**

Gender (male/female)

Personal identification number (year born)

Social status (Live alone/ Live with others)

Self-reported symptoms (scale from 0-10) as experienced at start of program (Pain at rest/ Pain in activity/ Depressive mood/ Anxiety)

Educational level (Elementary school/ Upper secondary school (up to 12 years)/ University degree up to 4 years/ University degree more than 4 years)

Did the program provide a person who tailored or coordinated your services? (Yes/ No/ Do not know)

**Questions for providers:**

To what degree have the following professionals been involved in the intervention provided for this patient? (Medical doctor/ Physical therapist/ Nurse/ Nutritionist/ Psychologist/ Vocational consultant/ Social worker/ Occupational therapist/ Pedagogue/ Work instructor/ Others)

Which of the interventions the patient received did the provided coordinator coordinate? (Your service/ Specialized health care/ General Practitioner/ Community based health care/ Workplace/ Social Insurance (NAV)/ Occupational Health Services/ Other interventions)

Who provided a coordinator? (Your service/ Specialized health care/ General Practitioner/ Community based health care/ Workplace/ Social Insurance (NAV)/ Occupational Health Services/ Other interventions)

To what extent have there been contact with other instances regarding this patient? (General Practitioner/ Social Insurance consultant (NAV)/ Leader/supervisor /Specialized health care/ Occupational Health Services/ Family/ Community based health care/ Work-life center (NAV arbeidslivssenter)/ Others)

Did the intervention directed toward this particular patient include the following adaptations? (No adaptations/ Adaptation of home/ Adaptation of leisure/ Adaptation of physical work environment/ Adaptation of psychosocial work environment/ Adaptation of work tasks/ Adaptation of work time)
